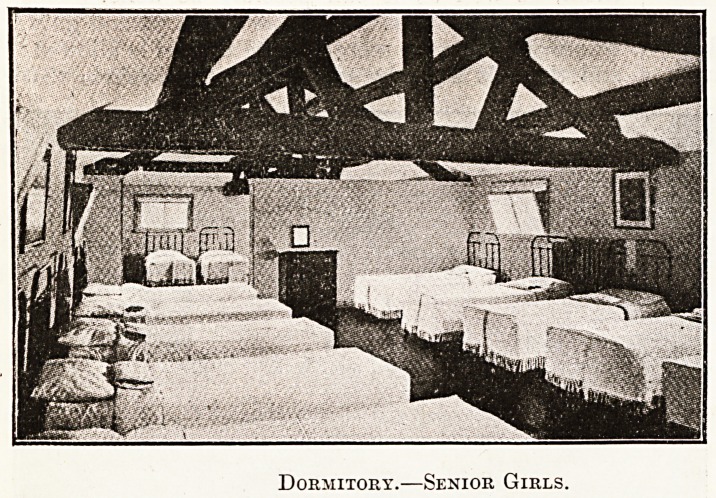# The Feeble-Minded as Producers

**Published:** 1913-10-11

**Authors:** 

**Affiliations:** Hon. Sec. Lancs. and Cheshire Society for Permanent Care of the Feeble-Minded


### THE FEEBLE-MINDED AS PRODUCERS.

### Their Permanent Care in Industrial and Farm Colonies.

By Miss DENDY, Hon. Sec. Lancs. and Cheshire Society for Permanent Care of the Feeble-Minded.

The colony at Sandlebridge for the feeble-minded was founded nearly twelve years ago under the auspices of the Lancashire and Cheshire Society for the Permanent Care of the Feeble-Minded. The object of the society was to provide such care for feeble-minded children as would (1) render them happy and keep them good and safe, (2) prevent them from being a danger to society, and (3) make them as useful as their limited capacities would permit. In order to achieve these results it was felt that it would be necessary to take the children in hand at as early an age as possible, and whilst conducting their elementary education in accordance with the regulations of the Board of Education, under whose certificate the school is carried on, to bear always in mind that it was desirable that in the future each child should be able to take up some useful occupation, and that it was therefore necessary to pay special attention to the training of hand and eye.

It should be understood clearly that the feeble in mind, no matter how carefully they are trained, will never be able to support themselves. The cost of the careful supervision which is always necessary makes it impossible that any colony should be run solely on the proceeds of the work done by the colonists. What can be done is, by careful adjustment of administration and by wise apportionment of work, according to the powers and wishes of the boys and girls cared for, to greatly reduce the sum which must be found either from the rates or from private philanthropy. The farm colony is probably much the best form of permanent care for the feeble-minded, both because work on the land does not necessarily mean the establishing of a protected trade, in competition with outside labour, and because of the great variety of work which is provided by the cultivation of the land. Other trades should be run in connection with the farming and gardening, so that by degrees the boys and girls may do all, or nearly all, of their own work. The girls, for whom quite separate provision should be made, may be occupied in doing housework and in washing, and making, and mending for the colony. I may say here, that our experience goes to show that, if a laundry be properly managed, there is no hardship in its work being done by the girls. They like the washing and ironing, the folding and mangling, the hanging out of the clothes, etc., as much as the boys like their work on the land. There is very considerable variety of occupation in a laundry.

At Sandlebridge we are beginning to reap the advantages of the scheme of training we have pursued. Of the two hundred and sixty-seven boys and girls now under our care, nearly one hundred are grown-up; that is, they are as much grown-up as they ever will be. Some three-fourths of the cases are boys and young men, and all were admitted to the schools under the age of thirteen. There are about one hundred and eighty school places. All the little children are regularly at school, learning, so far as their capacity permits, the usual school lessons in the morning, but in the afternoon occupied with manual work or in singing and games. They all learn to knit and to sew, boys and girls. All the vests and stockings used in the colony are made by the children. Of course, hand-knitting would never overtake the need for new stockings. But several of the girls have learned to use a knitting-machine, and one of these girls can turn out six pairs of stockings in a day, and that without working specially hard or for long hours. Very fine and strong baskets of cane and raffia are also made in school, the children working to patterns and doing the work, many of them, as well as it can possibly be done. This work has a great fascination for the boys and girls, who would give far more time to it than is allowed; and there is a ready sale for all the baskets that can be made. Very handsome rugs, in complicated patterns, are also made, and are again a great pleasure to the children. Younger boys and girls make baskets of plaited straw, and trays are made of basket-work and wood, which are strong and useful. The school-children, boys and girls, keep the gardens immediately round the school in order. It may be noted that the school-house is apart from all the dwelling-houses, so that scholars and teachers have to go out of doors to reach their daily work—a most important factor in keeping them strong and well. So soon as the children come to us the matrons of their different houses begin to teach them to be useful. That may sound hard, but is not so in reality seeing that the first years of training are devoted to learning to do what normal children do almost without teaching.

Warford Hall—Farm Yard.

Sometimes it takes many months to teach a child to fold its own clothes and wash its own hands. Good table-manners require much instruction; helpfulness from the older to the younger scholars has to be learned. From such things as these they proceed to blacking their own boots, putting away and getting out their Sunday clothes, and, presently, as they grow older and stronger, to cleaning taps and polishing floors. Some of the boys and girls never, no matter how old they are, get beyond these very elementary occupations. But then floors must be polished and taps cleaned, and the fact that the low-grade cases can do this releases others of higher intelligence for more advanced work. The great thing is to find out what each child can do and likes doing, and to remember that, if a child have the use of its limbs at all, it can be trained to do something. We must look in each case, not for what is abnormal in the child, but for what is normal. Everybody who has had much to do with the feeble-minded knows that they are far more remarkable for the queer patches of intelligence we find in their darkened minds than they are for their general dullness and incapacity. It is those patches of intelligence upon which we must work—too often much time and labour are expended in struggling to teach the weak in intellect to do that which they can never perform with either pleasure or skill, instead of discovering what their limited capacity can achieve and concentrating on that. By observing this rule we have been able at Sandlebridge to obtain a very fair composite mind for each group of workers. We have, as it were, pooled the various intelligences. So on the farm we have a group of lads who amongst them can do every part of the farm work. Only a few can milk—that is an occupation which demands more cleanliness than we can often secure from our boys. Still, enough are able for this task to carry it on with the help of the paid farmers. (And it is a big task, we never have fewer than thirty-two cows in milk.) Others can clean out the cow-houses, look after the horses, feed the pigs, use the chaff-cutter, flail, etc. Our crops are entirely got in by our own labour, and we find boys who could never do any arithmetic in school accurately weighing out potatoes and putting them in sacks for distribution to the various houses. Three very low-grade boys peel all the potatoes for the Colony with a machine —an arrangement which not only found occupation for lads who seemed almost useless, but incidentally saved us about three loads of potatoes a week. In the garden all the vegetables we use are grown, and not only that, but we sell some eighteen thousand plants in pot, chiefly geraniums and fuchsias, every year. About a year ago we started a cobbler's shop, and during that time the lads who are busy there have mended some five hundred pairs of boots and shoes. We look forward to making all our own boots presently, and doing our own tailoring. We do all our own carpentering, and the benches for the cobblers were made in the carpenter's shop. The head carpenter and his wife have charge of the men's house, and the young men there have made themselves a bowling-green, and put up a shed for skittles and for sitting in in wet weather. We do all our own plumbing, a couple of lads working regularly with the plumber.

Dormitory.—Senior Girls.

In each boy's house, two or three lads who are too under-sized or too delicate for hard work are kept as house-boys. We have found that the giving of a uniform quite makes up to them for their not being allowed to go to the farm or garden. There is wonderful virtue in red cloth and some buttons!

Our plans have not been less successful with the girls than with the boys. Almost without exception, they would choose to go to the laundry; but many of them are not strong enough for this work. Especially any of them who have weak eyes have to be kept away from the steam. Those who are not strong enough for washing learn housework and sewing. Some have to be kept to the rougher kinds of cleaning, not caring for any other. Others make good waitresses, and are useful in the kitchen. We have lately found it a very good plan to allow one or two of the young women to take special charge of a child who is more than usually helpless.

When our young men went into their new house every article of house-linen and the new shirts and night-shirts were made by the young women. The laundry-girls not only wash for our own Colony but also for some schools for epileptics near at hand. Their work is extremely good. We take great care that they are not at it too long. We have a trained nurse who, under the doctor, looks after all invalids and reports to him. Careful attention is paid to diet—we do not give much meat, but new milk and an abundance of fresh vegetables, butter and jam, and porridge.

We are just finishing the erection of a little hospital which will be a very great gain to the Colony, especially to nurse, who will be glad to have her invalids all collected under one roof.

We are, of course, but at the beginning of our work. We ought to do all our own work, even to the weaving of our own towelling, as is done at Waverley in Massachusetts.

## Figures and Tables

**Figure f1:**
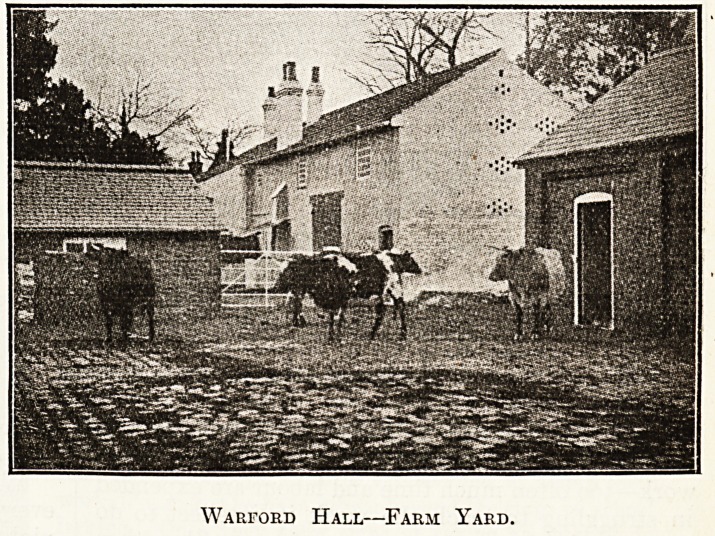


**Figure f2:**